# Dendrometers challenge the ‘moon wood concept’ by elucidating the absence of lunar cycles in tree stem radius oscillation

**DOI:** 10.1038/s41598-023-47013-y

**Published:** 2023-11-14

**Authors:** Jan Tumajer, Sabine Braun, Andreas Burger, Tobias Scharnweber, Marko Smiljanic, Lorenz Walthert, Roman Zweifel, Martin Wilmking

**Affiliations:** 1 Institute of Botany and Landscape Ecology, University of Greifswald, Soldmannstraße 15, 17487 Greifswald, Germany; 2 Department of Physical Geography and Geoecology, Faculty of Science, Charles University, Albertov 6, 12843 Prague, Czech Republic; 3 Institute for Applied Plant Biology AG, Benkenstrasse 254a, 4108 Witterswil, Switzerland; 4 Swiss Federal Institute for Forest, Snow and Landscape Research WSL, Zürcherstrasse 111, 8903 Birmensdorf, Switzerland

**Keywords:** Forest ecology, Astrobiology, Forestry, Plant physiology

## Abstract

Wood is a sustainable natural resource and an important global commodity. According to the ‘moon wood theory’, the properties of wood, including its growth and water content, are believed to oscillate with the lunar cycle. Despite contradicting our current understanding of plant functioning, this theory is commonly exploited for marketing wooden products. To examine the moon wood theory, we applied a wavelet power transformation to series of 2,000,000 hourly stem radius records from dendrometers. We separated the influence of 74 consecutive lunar cycles and meteorological conditions on the stem variation of 62 trees and six species. We show that the dynamics of stem radius consist of overlapping oscillations with periods of 1 day, 6 months, and 1 year. These oscillations in stem dimensions were tightly coupled to oscillations in the series of air temperature and vapour pressure deficit. By contrast, we revealed no imprint of the lunar cycle on the stem radius variation of any species. We call for scepticism towards the moon wood theory, at least as far as the stem water content and radial growth are concerned. We foresee that similar studies employing robust scientific approaches will be increasingly needed in the future to cope with misleading concepts.

## Introduction

Forests around the world are a rich source of diverse ecosystem services essential for human well-being and quality of life^1,2^. Many provided services can be economically evaluated and, consequently, traded as a commodity^3^. Although there is a deepening understanding of processes that affect the functioning and internal status of trees and how they scale up to determine wood features^4–8^, a few scientifically untested assumptions and popular beliefs about tree functioning are also spreading and might even be used to establish specific business models. For instance, according to the ‘moon wood theory’, the moon phase should tightly control the physiological processes of woody plants translating into systematic variation in wood quality^9–11^. Thus, stem and wood properties should follow a periodic oscillation pattern coupled with a synodic period of the moon, i.e., with a lunar cycle of on average 29.53 days. The effect of the moon on tree functioning should peak during the last quartile of the synodic period, i.e., between the third quarter and the new moon phase, when the moon is waning. In this interval, the tree stem should contain less water since it should be redistributed to the roots due to the alteration of sapflow and membrane activity induced by the moon^10^. Consequently, wood samples cut in different moon phases should undergo a different drying process, which should translate into different physical features^9^. Specifically, the timber produced from trees felled during the last quartile of the lunar cycle should have higher physical density resulting in superior properties including resistance to insect and fungi infestation, less cracking, higher aesthetic value thanks to specific colouring, and even reduced flammability^12^.

Although the moon wood theory contradicts the current mechanistic understanding of tree functioning, it is widespread in rumours and forestry practices in various regions of the world^10,12^. Moreover, it is often used to justify a higher price for timber and products made of wood harvested during the last quartile of the lunar cycle. Therefore, a rigorous assessment of its assumptions using robust datasets is needed to examine or challenge its validity. The previous studies that aimed to verify the moon wood theory mostly relied on sampling of wood during different phases of the lunar cycle and comparing specific properties between samples. They usually found non-significant or weak and unclear effects of the lunar cycle on wood properties^10,13^. The results of some studies even contradicted the assumption of the most intensive shrinkage and the highest wood density during the last quartile of the synodic period postulated by the moon wood theory^9^. However, the reliability of such studies might be limited due to a small number of sampling times in relation to the lunar cycle increasing the possibility of obtaining spurious relationships^9^, different parameters of trees and stands sampled in different lunar phases^14^, and a small number of sampled trees. In this regard, continuous high-frequency monitoring of the stem size variation using dendrometers presents an alternative approach for testing the moon wood theory, since it is not affected by any of the previously mentioned limitations and represents an established tool to reveal pulses and cycles in tree growth dynamics^15,16^. Moreover, dendrometer records are highly sensitive to the amount and state of the water inside the stem^17,18^ which in the moon wood theory is deemed to be a mechanistic link between the lunar cycle, stem physiology, and wood mechanical properties^10,12^.

**Figure 1:**
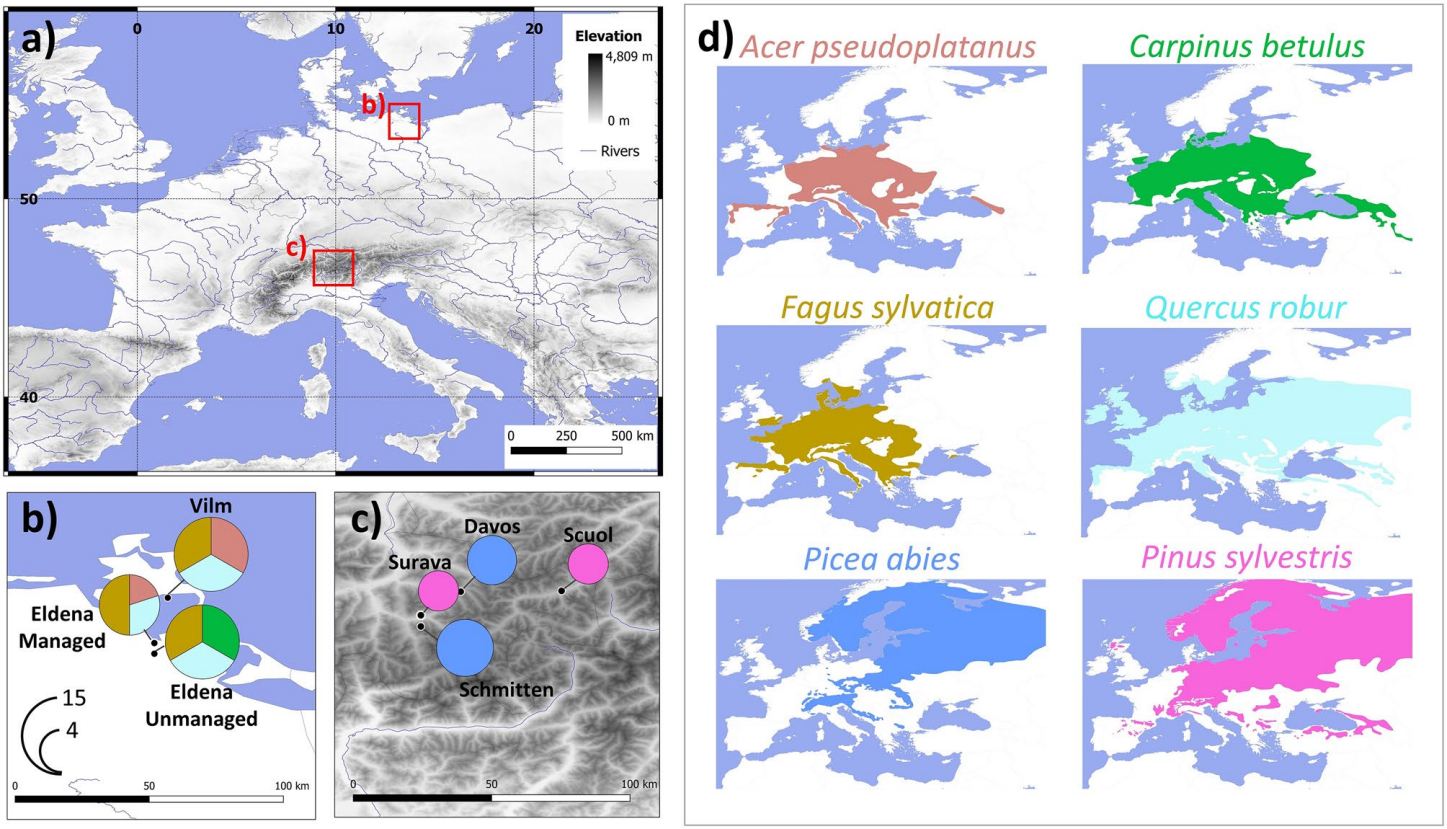
Position of seven monitoring plots equipped with dendrometers in Europe (**a**) with their species composition (**b**,**c**) and the potential distribution range of each species included in the study (**d**). The area of pie charts in (**b**,**c**) is proportional to the total number of trees equipped with dendrometers at a given plot in 2020—the scale shown in (**b**) applies also to (**c**). The colours of sectors in pie charts in (**b**,**c**) refer to colours used in (**d**). Species distribution ranges in (**d**) are according to^47^.

In this study, we used six years of high-frequency monitoring of stem radial growth and tree water deficit, both derived from automatic point dendrometer measurements^19^, for four temperate broadleaves and two boreal conifers to examine the assumptions of the moon wood theory (Fig. 1). The dataset comprised over 2,000,000 observations of stem size variation of 62 trees recorded in a 1-h timestep. To test the crucial assumption about the lunar cycle imprint on stem conditions we applied wavelet power transformation to the series of radial growth and tree water deficit. The wavelet power transformation is an established statistical tool that quantifies the importance of cycles with different periodicities by means of wavelet power statistics, i.e., a measure of the strength of a particular frequency component within a time series^20^. We statistically compared the cycles observed in series of radial growth and tree water deficit with the lunar cycle and local meteorological variables. Next, we used dendrometer data to test whether the stem water content varies within the lunar cycle. To do this, we quantified systematic offsets in mean tree water deficits and stem growth rates between the last quartile of the lunar cycle, i.e., the period of the most intense lunar effects on tree stems according to the moon wood theory^12^, and the first three quartiles of the lunar cycle.

## Results

**Figure 2:**
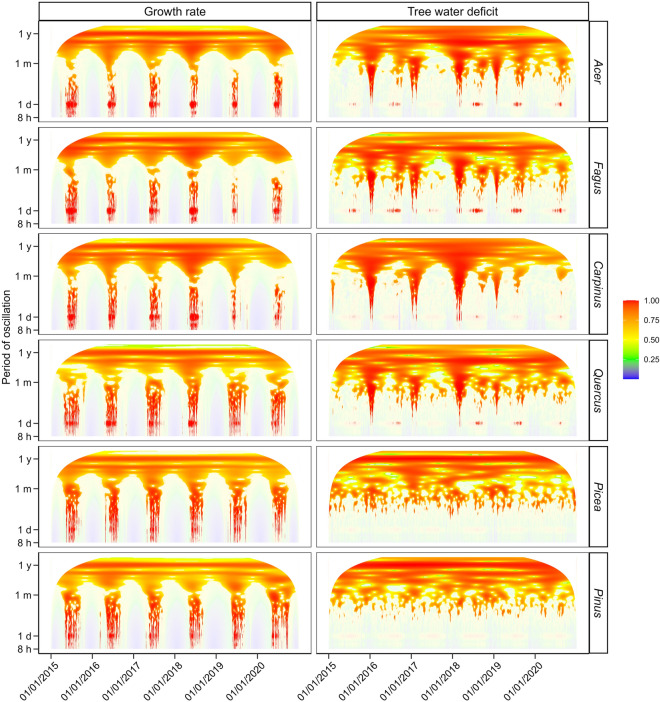
The wavelet power spectrum of periodic oscillations in radial growth rate (left column) and tree water deficit (right column) for four broadleaved and two coniferous species (rows). X-axes of the matrix indicate the 1-h timestep and Y-axes of the matrix indicate the period of wavelet oscillation (*h* hour, *d* day, *m* synodic month ≈ 29.53 d, *y* year). The colour gradient represents the standardized (0–1) power of the wavelet with red and blue colours indicating oscillations with the highest and lowest power for given species and variable, respectively. The colour of pixels with an estimated p-value > 0.05, i.e., non-significant wavelet power, is semitransparent.

**Figure 3:**
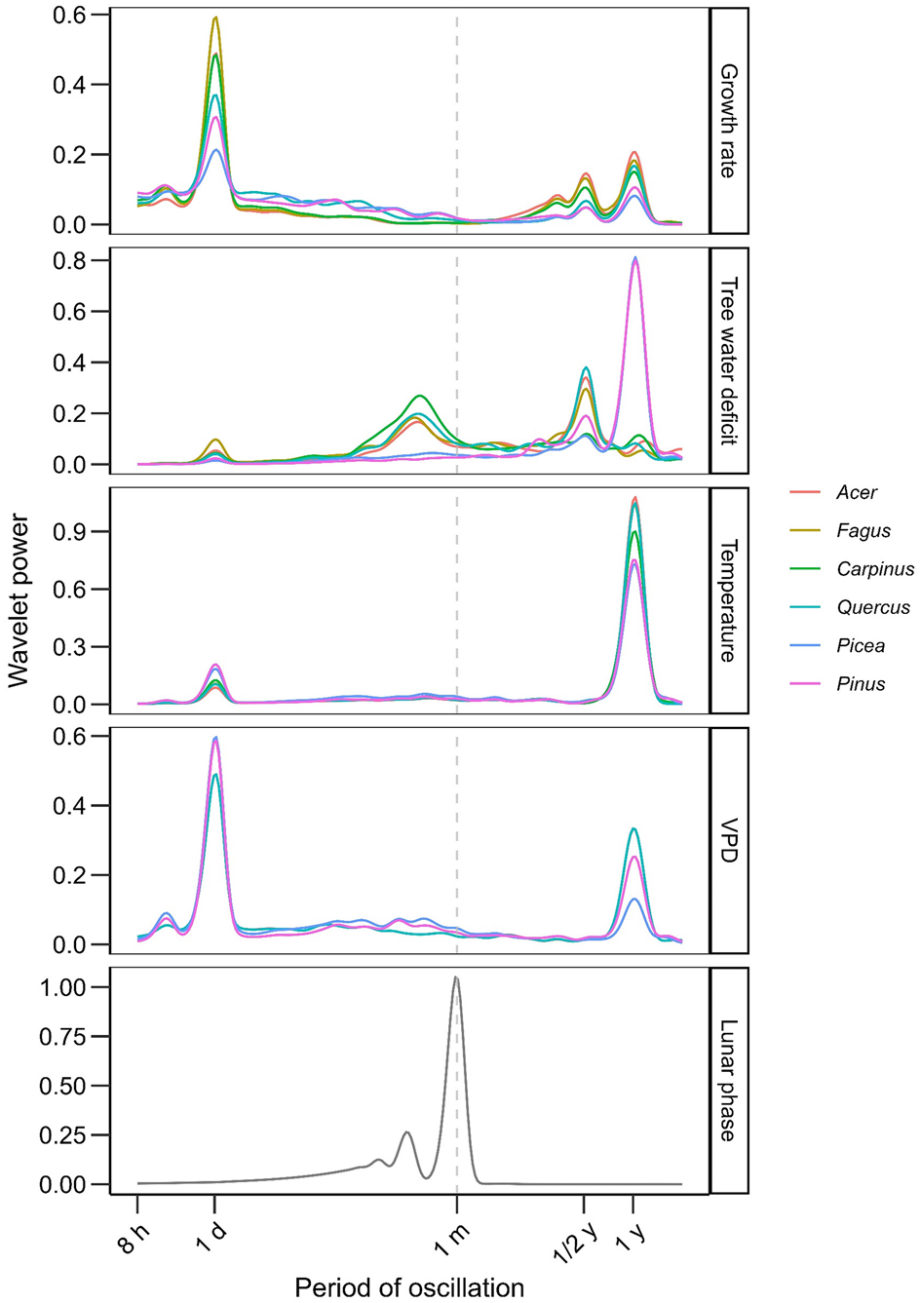
Mean power of wavelets with different oscillation periods in radial growth rate, tree water deficit, meteorological variables, and lunar phase averaged from January 2015 to December 2020. *h* hour, *d* day, *m* synodic month ≈ 29.53 d, *y* year. *VPD* vapour pressure deficit.

We used hourly dendrometer data for six tree species over the 2015–2020 period to calculate the time series of tree water deficit (TWD; variable sensitive to stem shrinkage due to variation in stem water content) and radial growth rate (GRO; variable sensitive to stem increment due to cambial activity). To highlight periodic oscillations in both variables, we converted the TWD and GRO series for each species into their wavelet power spectra. Wavelet powers of both TWD and GRO series showed systematic patterns with a high similarity between species (Fig. 2) and between sites (Supplementary Figs. S1-S2). Both types of series were composed of oscillations with a period of 1 day, 6 months, and 1 year. While the high power of annual and half-year cycles was stable over time, the pronounced influence of daily cycles was restricted to growing seasons. Daily cycles followed by annual and half-year cycles had the highest power in the GRO series of all species (Fig. 3). For TWD, the mean wavelet power was the highest for the annual cycle (conifers) or half-year cycle (broadleaves) followed by the daily cycle. Cycles with a period of approximately 14 days showed moderate importance in the TWD series of broadleaved species. However, their effect disappeared if TWD during timesteps with air temperature below 0 °C was equalised before the wavelet transformation (Supplementary Fig. S3). The mean power of cycles with a period of one synodic month was marginal both in the TWD and GRO series.

The wavelet power spectra of the GRO series showed a high visual (Fig. 3) as well as statistical (Table 1) similarity to the wavelet power spectrum of vapour pressure deficit. Excluding vapour pressure deficit from the generalised additive model of mean wavelet powers in the GRO series dramatically reduced the explained variability (R^2^ of the full model = 0.84, ΔR^2^ due to predictor exclusion = − 0.44). The power spectra of the TWD series were tightly linked to the wavelet power spectrum of air temperature with a major contribution of this meteorological variable to explained variability (R^2^ = 0.49, ΔR^2^ = − 0.31). By contrast, there was no visual similarity between the power spectrum of the lunar cycle and the GRO or TWD series. While the power spectra of GRO, TWD, and meteorological variables showed daily, half-year, and annual peaks, the lunar cycle was characterised by a single maximum of one synodic month with marginal interference peaks with a period equal to 0.5 and 0.25 synodic months. Moreover, the contribution of the lunar cycle to the predictive power was marginal for models of both GRO and TWD series (both ΔR^2^ < − 0.02). The neglectable contribution of the lunar cycle to R^2^ of the generalised additive models was independent of the choice of the mother wavelet and non-dimensional parameter used in the wavelet power transformation (Supplementary Table S1).

**Table 1:** Coefficients of determination (R^2^) for generalised additive models explaining wavelet powers in growth rate and tree water deficit series by respective wavelet powers in meteorological variables and lunar cycle.

	Growth rate	Tree water deficit
Full model	0.84	0.49
Excluding temperature	0.78 (− 0.06)	0.18 (− 0.31)
Excluding vapour pressure deficit	0.40 (− 0.44)	0.37 (− 0.12)
Excluding lunar phase	0.82 (− 0.02)	0.47 (− 0.01)

The negative values in parentheses indicate the difference in coefficients of determination of the full model and the model excluding the given variable (ΔR^2^). The calculations are based on the Morlet mother wavelet with k_0_ = 6. See the sensitivity analysis to the choice of the mother wavelet in Table S4.

**Figure 4:**
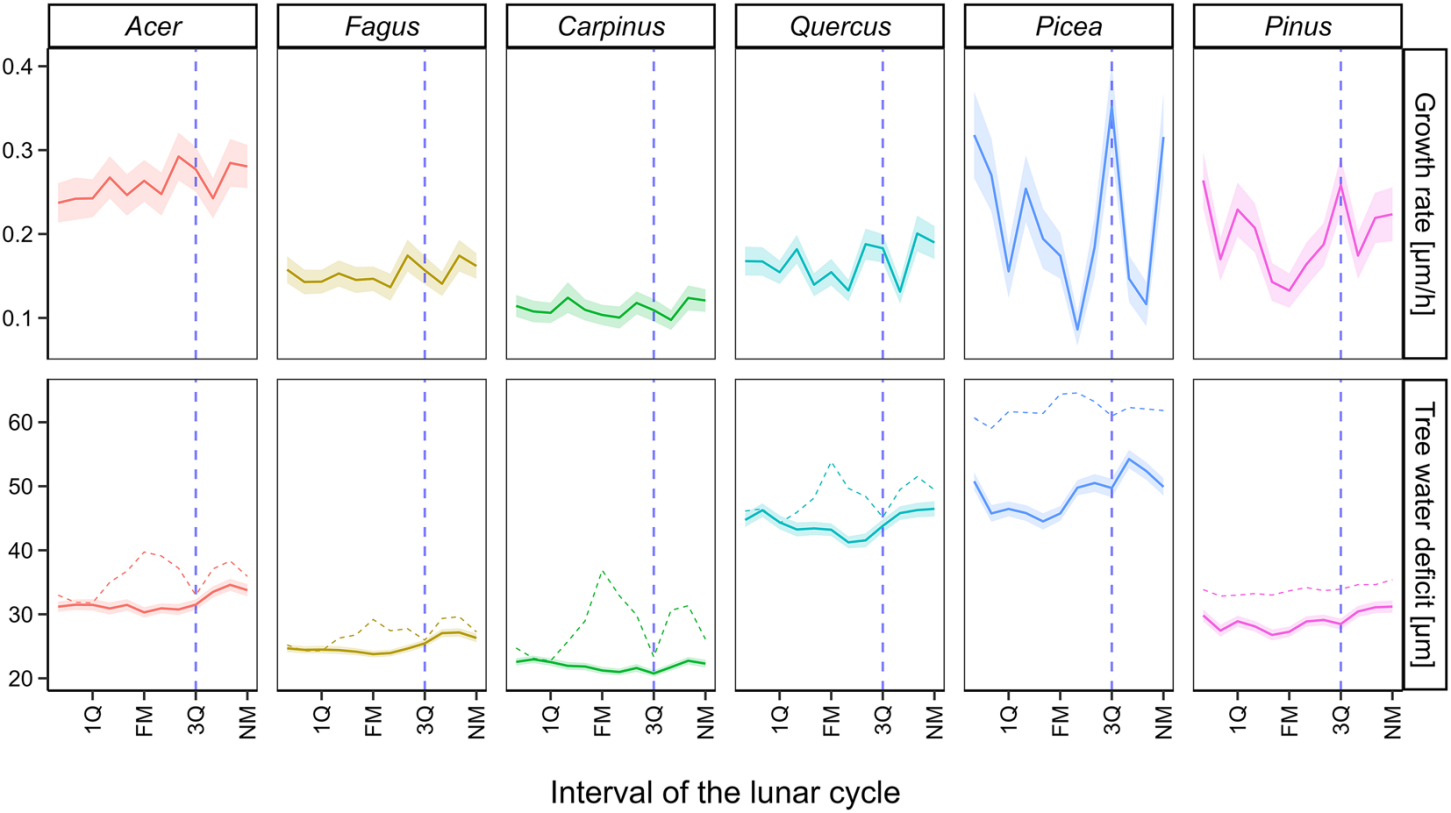
Mean radial growth rate (top row) and tree water deficit (bottom row) averaged for 12 intervals of the lunar cycle. Vertical dashed lines delimit an interval of the lunar cycle postulated by the moon wood theory to be optimal for tree harvesting due to the altered amount of water inside the stem (from 3Q to NM). Lines indicate mean values and ribbons 95% confidence intervals for a given phase of the lunar cycle. Dashed and solid lines of tree water deficit were determined based on all dendrometer observations and only on observations from timesteps with the air temperature above 0 °C, respectively. The data on the tree water deficit of coniferous species were multiplied by 0.2 to aid a direct visual comparison of the series among species on the same scale. *NM* new moon, *1Q* first quarter, FM full moon, *3Q* third quarter.

We calculated an angle defining the position of the moon around the orbit in 1-h resolution and converted its value into 12 equal intervals. However, the mean values of both GRO and TWD series showed no systematic variability in relation to these intervals of the lunar cycle for any of the species (Fig. 4). Excluding freezing periods resulted in the stabilization of mean TWD close to constant for the entire lunar cycle. The lack of monthly variability in mean values of the GRO and TWD series contrasts with their systematic annual (Supplementary Fig. S4) and daily patterns (Supplementary Fig. S5). The mean values of GRO and TWD over the entire dataset equaled 0.18 µm h^−1^ and 97.81 µm, respectively. The highest standard deviation of GRO and TWD series was observed between days of the year for both variables (mean across all species of 0.33 µm h^−1^ and 56.66 µm, respectively) followed by variability between hours of the day (0.13 µm h^−1^ and 5.57 µm) towards the minimum over the course of the lunar cycle, i.e. between 12 moon positions around the orbit (0.03 µm h^−1^ and 3.70 µm).

## Discussion

Our results highlight that periodic oscillations of stem radial growth and tree water deficit of temperate and boreal tree species are governed by cycles linked to daily and seasonal variations in meteorological conditions. By contrast, the coupling of stem size to the lunar cycle–as assumed by the moon wood theory–was not significant. According to the wavelet power transformation, oscillations with a period of one synodic month showed minimal power and neglectable contribution to the variability of radial growth and tree water deficit. The prominent cycles in stem growth and water content were rather tightly linked to variations in vapour pressure deficit and air temperature. Moreover, we observed no systematic changes in mean stem growth rate and water status within the lunar cycle. The last quartile of the synodic period, i.e., the interval with a strong moon effect on stem water content postulated by the moon wood theory^12^, showed similar rates of radial growth and stem shrinkage as the other intervals of the synodic period. Therefore, our results confirmed the importance of meteorological variability for the radial growth and tree stem status on various temporal scales ranging from sub-daily^15^ to seasonal^21^ and inter-annual^22^. Thus our findings support an earlier scientific scepticism about the moon's effects on the physiology of woody plants^10,11,23^ as none of our analyses revealed a significant imprint of the lunar cycle on stem growth or stem water content.

Systematic patterns of wavelet power observed for all species showed that stem size variation is a periodic process characterised by overlapping high and low-frequency oscillations. High-frequency oscillations are represented by cycles with a period of 24 h linked to diurnal stem shrinkage and swelling and nocturnal radial growth^15^. Stem water content tends to be lower during the day when the tree transpires and higher during night times when stomata are closed and the daytime water losses are refilled. During the growing season, this cycle is controlled by transpiration through its effect on turgor pressure inside cambial cells^24^. Besides, prominent daily cycles of tree water deficit might be driven by winter freezing and melting of intercellular water^18,25^. Low-frequency cycles with a period of one year reflect the restriction of cambial activity only to the warm seasons typical for temperate and boreal climates^26–28^. The intermediate cycle with a period of half a year might be a consequence of similar meteorological conditions and stem dynamics in spring and autumn, respectively the physiological changes between the active and the dormant phase in spring and autumn^29^. In contrast to these three cycles, which are associated with systematic oscillations of meteorological conditions, we found no significant monthly variability in either radial growth or tree water deficit series. If the process of stem shrinkage followed a monthly oscillation pattern, tree water deficit as a variable sensitive to stem water content^30^ should show a high power for a wavelet with a period of lunar cycle. However, the weak power of monthly oscillations in a series of growth rates and tree water deficit was stable over time and consistent among species and sites. Consequently, the variability in mean wavelet powers of growth rate and tree water deficit was largely explained by meteorological series with a neglectable contribution of the lunar cycle.

In addition to the non-significant contribution of the lunar cycle to wavelet patterns, we observed no effect of the lunar cycle on mean values of growth rate and tree water deficit. Most importantly, mean rates of radial growth and tree water deficit did not show systematic differences between the last quartile and the first three quartiles of the synodic period. According to popular beliefs associated with the moon wood theory, the last quartile of the synodic period is a pivotal interval for both forestry^12^ and agriculture^10^ . During this period, the effect of the moon on plants should lead to a strong stem shrinkage due to altered sapflow. The lack of variability in mean growth rate and tree water deficit along the lunar cycle contrasts with prominent and systematic cycles of both variables observed over the year and during the day. The standard deviation of tree water deficit over the course of the lunar cycle equalled on average only 3.70 µm which represents less than 4% of the long-term mean. By contrast, standard deviations related to both annual and diel variability were higher and accounted for 58% and 6% of the long-term mean, respectively. This challenges the essential assumption of the moon wood theory about the systematic offset of the stem water content during the last quartile from the rest of the lunar cycle^10,12^.

Previous studies verifying the effects of the lunar cycle on plant growth were mostly limited by insufficient temporal resolution, small replication, and invasive or destructive sampling distorting repeated observations^9,14^. In this study, we overcame these limitations by using data from low-invasive high-frequency monitoring of stem size variation covering six years, six tree species, and 62 trees. Using continuous dendrometer records we assessed the influence of 74 consecutive lunar cycles between January 2015 and December 2020 on stem dimensions, including actual growth and radius changes due to variations in stem water content. Nevertheless, our approach has its limitations. First, dendrometer data provide precise information about stem water content and radial growth dynamics, but not about other physical features of the wood postulated to be sensitive to the lunar cycle by the moon wood theory, e.g., wood density, colour, flammability, or decay resistance^12^. These features, whose assessment would require highly invasive or even destructive sampling^9^, are independent or only indirectly linked to stem radius and, therefore, dendrometers provide limited insight into their variation^8^. Moreover, the winter mean values of tree water deficit in broadleaves were affected by severe stem shrinkage in response to sporadic intercellular water freezing^18^. We suggest that irregular occurrence of winter freezing in relatively warm Baltic lowland might be responsible for a high power of approx. 14-day wavelets in broadleaves. Therefore, we effectively removed these cycles by equalising tree water deficit during timesteps with air temperature below 0 °C. The effect of the lunar cycle on stem shrinkage was neglectable both in the original dataset as well as in a dataset with artificially removed cycles from freezing periods. This further confirms that stem dimensions respond rather to concurrent meteorological conditions^4,31^, including winter air temperature^18^, than to the lunar cycle. Our study applied established approaches of empirical monitoring of the stem size variation and statistical processing of dendrometer data. Therefore, our conclusions need to be interpreted as a lack of statistical support for the significant imprint of the lunar cycle on the stem size oscillation, but not as a piece of ultimate evidence for the non-existence of these mechanisms. Nevertheless, our results unequivocally support previous correlative studies^13,14,32,33^ and meta-analyses^10,11^ demonstrating the absence of lunar effects on the growth and fitness of living organisms. In the future, it is desirable to apply a similar approach to dendrometer series from tropical and subtropical environments to better communicate scepticism towards the moon wood theory on a global scale.

## Methods

### Study sites and species

We monitored the stem size variation of 62 trees of six Palearctic tree species using automatic point dendrometers from 1st January 2015 to 31st December 2020. Therefore, the monitoring covered 74 consecutive lunar cycles. Species involved in the monitoring included two conifers with a mainly boreal distribution (*Picea abies* (L.) Karst., *Pinus sylvestris* L.), three temperate diffuse-porous broadleaves (*Fagus sylvatica* L., *Acer pseudoplatanus* L., *Carpinus betulus* L.) and one temperate ring-porous broadleaf (*Quercus robur* L.). Among the species under study, *P. abies* is the most frequently harvested to produce moon-wood timber and pile wood, while various broadleaves are often used for moon-wood furniture and ornamental crafting^12^. The stem size variation of broadleaves was monitored at three plots situated within the distance of 30 km in the Baltic lowland, Northern Germany (Fig. 1; centre of the region 54.077°N, 13.455°E)^26,34^. The dendrometer data for conifers were obtained from four plots of the TreeNet network located within a 50 km radius in the eastern Alpine region of Switzerland (46.765°N, 9.852°E)^35^. While the region with dendrometer monitoring of broadleaves has a temperate humid climate with mild winters and warm summers, conifer plots are located in a cold temperate zone with systematic variability of climatic conditions along the elevational gradient (Supplementary Fig. S6).

### Dendrometer monitoring of stem size variation

We installed automatic point dendrometers to monitor the variation of the stem radius of 5–15 dominant, healthy, and mature trees per species (Fig. 1). Point dendrometers DR-1 (Ecomatik, Germany), ZN11-T-IP, and ZN12-T-WP (Natkon, Switzerland) were used to record radius changes in 10 to 30-min timesteps. To ensure consistency, we subsampled all dendrometer data into the 1-h interval. The instrumentation of the first dendrometers was performed in 2013 and 2011 in Germany and Switzerland, respectively. Since then, the number of dendrometers progressively increased leading to a sufficient replication of at least 5 monitored trees per species during the entire 2015–2020 period (Supplementary Table S2). The installation of point dendrometers at each of the plots was permitted by forest owners and complied with all relevant legislations and guidelines of LTER-D and TreeNet monitoring networks.

The stem size variation recorded by dendrometers represents a cumulative effect of irreversible stem size increment due to cambial growth and reversible stem size oscillation due to the changing amount and state of water inside the stem^7,18^. To separate irreversible stem size increments from reversible swelling and shrinking, we used the ‘zero growth’ statistical approach^36^. The approach assumes that the entire variability of the stem radius below its previous maximum is a consequence of reversible processes driven by alteration of stem water content, while any increment of stem radius exceeding the previous maximum is driven by irreversible growth. The intensity of stem shrinkage during periods with stem diameter below the previous maximum can be quantified using a variable called ‘tree water deficit’ (TWD, μm). TWD is defined as the difference between concurrent stem diameter and its previous maximum. High values of TWD indicate strong stem shrinkage often driven by intensive transpiration^15,17,31^ or intercellular water freezing^18^. Next, the ‘growth rate’ variable (GRO, μm h^−1^) is based on the stem size increment on top of the previous maximum during periods when the previous maximum is exceeded by concurrent stem size. GRO reflects a pace of stem radial growth which in temperate and dry climates occurs episodically under favourable meteorological and hydropedological conditions^4,37^. Since trees do not grow during periods of stem shrinkage^24,36^, each timestep of each tree can be characterised by a nonzero value of either TWD or GRO, but not both. We calculated the time series of TWD and GRO with the 1-h resolution for each tree and, subsequently, averaged them into mean TWD and GRO series per species. Unrealistic values of GRO exceeding 100 μm h^−1^ at the tree level probably due to dendrometer manipulation or technical issues were replaced with 0 μm h^−1^. Moreover, missing values in the TWD and GRO series at the species level were replaced with a long-term median for a given species. However, both types of issues in the data series were rare and affected only 1,532 of more than 630,000 timesteps (< 0.25%) of the mean GRO and TWD series of six species.

Along with the stem size variation, we also measured various meteorological variables including air temperature and relative air humidity at each site^26,35^. We used both meteorological variables to calculate the time series of vapour pressure deficit in a 1-h resolution^38^. Our motivation to include vapour pressure deficit in the analysis reflected growing evidence of air humidity's significance in controlling the daily and yearly growth of trees in forest ecosystems^39^.

### Statistical assessment of cycles in tree water deficit and growth rate

We used wavelet power transformation to highlight periodic oscillations in the mean TWD and GRO series for each species. Wavelet power transformation represents a mathematical convolution capable of decomposing time series into a time–frequency scale^40^. Therefore, it is a suitable tool to identify dominant frequencies of variability in series with a high level of non-stationarity including dendrometer data^23,41^. We generated 221 wavelets, i.e., wave-like oscillations, derived from the Morlet mother wavelet with non-dimensional parameter k_0_ = 6. The frequencies of individual wavelets varied between 8 h (8 consecutive timesteps with a resolution of one hour) and 1.9 years (16,962 consecutive timesteps; Supplementary Table S3). The Morlet mother wavelet was used for its suitability for analysing time series with expected smooth and continuous variation in wavelet amplitude. Compared to the other mother functions, it has a high frequency localization at the expense of lower time localization^20^, which is appropriate for later steps of our analysis. Next, we shifted each wavelet over time from the first (1st January 2015 00:00) to the last (31st December 2020 23:00) timestep of the dendrometer series. At each position, we calculated a variable called ‘wavelet power’ which quantifies how much each wavelet is in phase with observed series of TWD and GRO. Since we aimed to compare wavelet power spectra of different variables and species, we pre-processed TWD and GRO series of each species by subtracting the mean and dividing by the standard deviation^20^.

We plotted a matrix showing wavelet powers of different frequencies and their evolution over time for each species and TWD and GRO series. Next, we averaged the power of each wavelet across the entire monitoring period into the global wavelet spectrum and plotted them as line charts to assess the long-term importance of specific oscillations in the GRO and TWD series. We used both charts to compare the power of wavelets with scientifically evidenced oscillations in stem dimensions, i.e., daily^15^ and annual^34^ cycles, with monthly cycles assumed by the moon wood theory.

### External drivers of oscillations in tree water deficit and growth rate

Similar to the GRO and TWD series, we performed wavelet power transformation also for the time series of meteorological variables at the species level and the lunar cycle. Meteorological variables included air temperature and vapour pressure deficit recorded in hourly resolution. We subsampled the meteorological series to contain for each species only the periods with at least two years without any segment of missing data longer than six hours (Supplementary Table S4). The remaining gaps were rare and were replaced by long-term medians to produce continuous series without any missing data required by wavelet power transformation^40^. Next, we calculated the synodic position of the moon indicating its angular position around the orbit for each timestep with a 1-h resolution^42^. Similar to the GRO and TWD series, climatic variables and lunar cycle were standardized before calculating wavelet power transformation. From the power spectrum of each meteorological variable and the lunar cycle, we calculated the mean long-term power of each wavelet. We built generalised additive models explaining the variability in mean powers of specific frequencies in GRO and TWD series by their respective powers in series of temperature, vapour pressure deficit, and the lunar cycle. The models were constructed as follows:

$$\left.\genfrac{}{}{0pt}{}{{GRO}_{(f;2015-2020)}}{{TWD}_{(f;2015-2020)}}\right\}={a0}_{SPE}+s\left({TEMP}_{\left(f;2015-2020\right)}\right)+s\left({VPD}_{\left(f;2015-2020\right)}\right)+ s\left({LUN}_{\left(f;2015-2020\right)}\right),$$

where GRO, TWD, TEMP, VPD, and LUN stand for mean wavelet powers of growth rate, tree water deficit, air temperature, vapour pressure deficit, and the lunar cycle, respectively, a0 is regression intercept specific for individual species, f is the frequency of the wavelet (Supplementary Table S2), and s is a thin plate regression smoothing term. We calculated the coefficient of determination for a full model (R^2^) and assessed the importance of meteorological and lunar variables by means of a difference between the coefficients of determination of a full model and the model excluding the given predictor (ΔR^2^).

### Sensitivity analysis of the wavelet power transformations

The wavelet power transformations of GRO, TWD, and meteorological variables were performed at the species level. This was motivated by the fact that wavelet power transformation requires continuous time series without the presence of missing values^40^ which, however, were common in GRO and TWD series at the tree level due to temporary failures of individual sensors. Nevertheless, to assess the between-site variability in oscillation patterns, we calculated wavelet power transformation for individual sites and sufficiently long segments of GRO and TWD without missing values. Since the results showed a high similarity of oscillations between sites (Supplementary Figs. S1–S2), we present only the results from the species level in the paper. Next, we assessed the sensitivity of our results to the arbitrary choice of the Morlet mother wavelet with non-dimensional parameter k_0_ = 6. To do so, we recalculated individual steps of our analysis including generalised additive models for three different types of mother wavelets (Morlet, Paul, Derivative of Gaussian) and varying non-dimensional parameters between 1 and 10. Finally, we also compared the wavelet power spectrum of the original and modified TWD series at the species level. The modification was based on replacing values of TWD during timesteps with air temperature below 0 °C by the long-term median of TWD. The later analysis was motivated by the possibly strong shrinking effect of freezing of intercellular water on stem dimensions^18^. Using constant values of TWD for freezing periods removed any oscillations associated with the irregular freezing occurrence.

### Variations of mean growth rate and tree water deficit concerning the lunar cycle

The synodic position of the moon was converted into 12 equal intervals where consecutive pairs of intervals 12–1, 3–4, 6–7, and 9–10 represent the new moon, first quarter, full moon, and third quarter, respectively. Next, we averaged TWD and GRO series for each species and each of the 12 synodic positions. We compared the mean values of TWD and GRO between the third quarter and the new moon phase with the rest of the lunar cycle since stems should significantly shrink during this period according to the moon wood theory^10,12^. Similar to the wavelet power transformation, we calculated mean values for each synodic position based on the original dataset of TWD as well as for the subsample unaffected by the freezing. For the latter, we subsampled timesteps with the air temperature above 0 °C. Finally, for each species, we compared the standard deviation of GRO and TWD between 12 intervals of synodic position to the standard deviations of these variables between days of the year (annual variability) and hours of the day (diel variability).

All steps of data processing were performed in R 4.1.2.^43^ using the packages ‘WaveletComp’^44^ (wavelet power transformation), ‘lunar’^42^ (synodic position of the moon), ‘plantecophys’^38^ (vapour pressure deficit), ‘mgcv’^45^ (generalised additive models), and ‘ggplot2’^46^ (charts' plotting).

### Supplementary Information

Supplementary Information.

## Data Availability

The datasets analysed during the current study are available in the GitHub repository (https://github.com/jantumajer/MoonWood).
