# Supplementary material for: Dendrometers challenge the ‘moon wood concept’ by elucidating the absence of lunar cycles in tree stem radius oscillation
Source: Sci Rep. 2023 Nov 14;13:19904. doi: 10.1038/s41598-023-47013-y (PMC10645754; doi:10.1038/s41598-023-47013-y)
Supplement: Supplementary file 1 — Supplementary Information. [file 41598_2023_47013_MOESM1_ESM.docx]

Dendrometers challenge the ‘moon wood concept’ by elucidating the absence of lunar cycles in tree stem radius oscillation

Jan Tumajer^12*^, Sabine Braun^3^, Andreas Burger^1^, Tobias Scharnweber^1^, Marko Smiljanic^1^, Lorenz Walthert^4^, Roman Zweifel^4^, Martin Wilmking^1^

1. University of Greifswald, Institute of Botany and Landscape Ecology, Soldmannstraße 15, 17487 Greifswald, Germany
2. Charles University, Faculty of Science, Department of Physical Geography and Geoecology, Albertov 6, 12843 Prague, Czech Republic
3. Institute for Applied Plant Biology AG, Benkenstrasse 254a, 4108 Witterswil, Switzerland
4. Swiss Federal Institute for Forest, Snow and Landscape Research WSL, Zürcherstrasse 111, 8903 Birmensdorf, Switzerland

[*tumajerj@natur.cuni.cz](mailto:*tumajerj@natur.cuni.cz)

**Supplementary section**

**Table S1:** Sensitivity analysis of generalised additive models explaining power spectra of growth rate and tree water deficit by power spectra of climatic variables and the lunar cycle to the choice of the wavelet mother function and non-dimensional parameter. The bold row highlights a model presented in the main body of the manuscript.

| **Wavelet mother** | **Non-dimensional**  **parameter** | **Growth rate** | | | | **Tree water deficit** | | | |
| --- | --- | --- | --- | --- | --- | --- | --- | --- | --- |
|  |  | **R^2^ of the full model** | **ΔR^2^ VPD** | **ΔR^2^ Temp** | **ΔR^2^ Lunar** | **R^2^ of the full model** | **ΔR^2^ VPD** | **ΔR^2^ Temp** | **ΔR^2^ Lunar** |
| Derivative of Gaussian ('DOG') | m = 1 | 0.73 | -0.14 | -0.02 | -0.05 | 0.68 | -0.12 | -0.12 | -0.02 |
|  | m = 2 | 0.79 | -0.17 | -0.05 | -0.07 | 0.63 | -0.09 | -0.14 | -0.01 |
|  | m = 3 | 0.84 | -0.22 | -0.05 | -0.07 | 0.61 | -0.10 | -0.16 | -0.01 |
|  | m = 4 | 0.86 | -0.24 | -0.04 | -0.05 | 0.58 | -0.10 | -0.19 | -0.01 |
|  | m = 5 | 0.86 | -0.25 | -0.05 | -0.05 | 0.57 | -0.10 | -0.20 | -0.01 |
|  | m = 6 | 0.86 | -0.26 | -0.05 | -0.04 | 0.54 | -0.10 | -0.21 | -0.02 |
|  | m = 7 | 0.86 | -0.30 | -0.06 | -0.04 | 0.53 | -0.10 | -0.24 | -0.01 |
|  | m = 8 | 0.85 | -0.32 | -0.06 | -0.04 | 0.52 | -0.10 | -0.25 | -0.01 |
|  | m = 9 | 0.85 | -0.34 | -0.06 | -0.04 | 0.51 | -0.10 | -0.26 | -0.01 |
|  | m = 10 | 0.85 | -0.37 | -0.06 | -0.03 | 0.50 | -0.10 | -0.26 | -0.01 |
| Paul | m = 1 | 0.83 | -0.09 | -0.02 | -0.04 | 0.72 | -0.17 | -0.16 | -0.06 |
|  | m = 2 | 0.87 | -0.23 | -0.01 | -0.03 | 0.63 | -0.15 | -0.16 | -0.02 |
|  | m = 3 | 0.88 | -0.24 | -0.04 | -0.04 | 0.61 | -0.11 | -0.17 | -0.01 |
|  | m = 4 | 0.88 | -0.29 | -0.07 | -0.06 | 0.61 | -0.12 | -0.21 | -0.02 |
|  | m = 5 | 0.87 | -0.32 | -0.09 | -0.05 | 0.61 | -0.11 | -0.27 | -0.02 |
|  | m = 6 | 0.86 | -0.28 | -0.10 | -0.04 | 0.63 | -0.13 | -0.31 | -0.02 |
|  | m = 7 | 0.86 | -0.27 | -0.09 | -0.03 | 0.65 | -0.13 | -0.34 | -0.02 |
|  | m = 8 | 0.86 | -0.26 | -0.09 | -0.03 | 0.66 | -0.13 | -0.37 | -0.02 |
|  | m = 9 | 0.86 | -0.26 | -0.08 | -0.03 | 0.70 | -0.12 | -0.41 | -0.02 |
|  | m = 10 | 0.86 | -0.27 | -0.07 | -0.03 | 0.67 | -0.13 | -0.37 | -0.02 |
| Morlet | k_0_ = 1 | 0.91 | -0.11 | -0.01 | -0.01 | 0.85 | -0.04 | -0.09 | -0.02 |
|  | k_0_ = 2 | 0.88 | -0.17 | -0.02 | -0.03 | 0.64 | -0.10 | -0.13 | -0.02 |
|  | k_0_ = 3 | 0.87 | -0.25 | -0.06 | -0.05 | 0.58 | -0.11 | -0.20 | -0.02 |
|  | k_0_ = 4 | 0.85 | -0.36 | -0.07 | -0.05 | 0.54 | -0.12 | -0.28 | -0.01 |
|  | k_0_ = 5 | 0.83 | -0.41 | -0.06 | -0.02 | 0.51 | -0.11 | -0.28 | -0.01 |
|  | **k_0_ = 6** | **0.84** | **-0.44** | **-0.06** | **-0.02** | **0.49** | **-0.12** | **-0.31** | **-0.01** |
|  | k_0_ = 7 | 0.84 | -0.42 | -0.08 | 0.00 | 0.48 | -0.10 | -0.33 | -0.01 |
|  | k_0_ = 8 | 0.83 | -0.42 | -0.07 | 0.00 | 0.50 | -0.10 | -0.38 | 0.00 |
|  | k_0_ = 9 | 0.83 | -0.32 | -0.07 | 0.00 | 0.50 | -0.10 | -0.38 | 0.00 |
|  | k_0_ = 10 | 0.84 | -0.31 | -0.08 | 0.00 | 0.51 | -0.09 | -0.43 | 0.00 |

VPD = vapour pressure deficit; Temp = air temperature; Lunar = lunar cycle

**Table S2**: Number of trees equipped with dendrometers per species and year

| **Species** | **2015** | **2016** | **2017** | **2018** | **2019** | **2020** |
| --- | --- | --- | --- | --- | --- | --- |
| *Acer pseudoplatanus* | 5 | 5-7^1^ | 7 | 7 | 7 | 7 |
| *Carpinus betulus* | 5 | 5 | 5 | 5 | 5 | 5 |
| *Fagus sylvatica* | 10 | 10-15^1^ | 15 | 15 | 15 | 15 |
| *Quercus robur* | 10 | 10-13^1^ | 13 | 13 | 13 | 13 |
| *Picea abies* | 14 | 14 | 14 | 14 | 14 | 14 |
| *Pinus sylvestris* | 8 | 8 | 8 | 8 | 8 | 8 |

^1^ – New dendrometers were assembled in April 2016

| **Wavelet** | **Frequency** | | | **Wavelet** | **Frequency** | | | **Wavelet** | **Frequency** | | | **Wavelet** | **Frequency** | | |
| --- | --- | --- | --- | --- | --- | --- | --- | --- | --- | --- | --- | --- | --- | --- | --- |
|  | **Hours** | **Days** | **Years** |  | **Hours** | **Days** | **Years** |  | **Hours** | **Days** | **Years** |  | **Hours** | **Days** | **Years** |
| 1 | 8 | 0.33 | 0.0009 | 57 | 57.68 | 2.4 | 0.0066 | 112 | 388.02 | 16.17 | 0.0443 | 167 | 2610.3 | 108.76 | 0.298 |
| 2 | 8.28 | 0.35 | 0.0009 | 58 | 59.71 | 2.49 | 0.0068 | 113 | 401.71 | 16.74 | 0.0459 | 168 | 2702.35 | 112.6 | 0.3085 |
| 3 | 8.57 | 0.36 | 0.001 | 59 | 61.82 | 2.58 | 0.0071 | 114 | 415.87 | 17.33 | 0.0475 | 169 | 2797.65 | 116.57 | 0.3194 |
| 4 | 8.88 | 0.37 | 0.001 | 60 | 64 | 2.67 | 0.0073 | 115 | 430.54 | 17.94 | 0.0491 | 170 | 2896.31 | 120.68 | 0.3306 |
| 5 | 9.19 | 0.38 | 0.001 | 61 | 66.26 | 2.76 | 0.0076 | 116 | 445.72 | 18.57 | 0.0509 | 171 | 2998.45 | 124.94 | 0.3423 |
| 6 | 9.51 | 0.4 | 0.0011 | 62 | 68.59 | 2.86 | 0.0078 | 117 | 461.44 | 19.23 | 0.0527 | 172 | 3104.19 | 129.34 | 0.3544 |
| 7 | 9.85 | 0.41 | 0.0011 | 63 | 71.01 | 2.96 | 0.0081 | 118 | 477.71 | 19.9 | 0.0545 | 173 | 3213.66 | 133.9 | 0.3669 |
| 8 | 10.2 | 0.42 | 0.0012 | 64 | 73.52 | 3.06 | 0.0084 | 119 | 494.56 | 20.61 | 0.0565 | 174 | 3326.99 | 138.62 | 0.3798 |
| 9 | 10.56 | 0.44 | 0.0012 | 65 | 76.11 | 3.17 | 0.0087 | 120 | 512 | 21.33 | 0.0584 | 175 | 3444.31 | 143.51 | 0.3932 |
| 10 | 10.93 | 0.46 | 0.0012 | 66 | 78.79 | 3.28 | 0.009 | 121 | 530.06 | 22.09 | 0.0605 | 176 | 3565.78 | 148.57 | 0.4071 |
| 11 | 11.31 | 0.47 | 0.0013 | 67 | 81.57 | 3.4 | 0.0093 | 122 | 548.75 | 22.86 | 0.0626 | 177 | 3691.52 | 153.81 | 0.4214 |
| 12 | 12.13 | 0.51 | 0.0014 | 68 | 84.45 | 3.52 | 0.0096 | 123 | 568.1 | 23.67 | 0.0649 | 178 | 3821.7 | 159.24 | 0.4363 |
| 13 | 12.55 | 0.52 | 0.0014 | 69 | 87.43 | 3.64 | 0.01 | 124 | 588.13 | 24.51 | 0.0671 | 179 | 3956.48 | 164.85 | 0.4517 |
| 14 | 13 | 0.54 | 0.0015 | 70 | 90.51 | 3.77 | 0.0103 | 125 | 608.87 | 25.37 | 0.0695 | 180 | 4096 | 170.67 | 0.4676 |
| 15 | 13.45 | 0.56 | 0.0015 | 71 | 93.7 | 3.9 | 0.0107 | 126 | 630.35 | 26.26 | 0.072 | 181 | 4240.45 | 176.69 | 0.4841 |
| 16 | 13.93 | 0.58 | 0.0016 | 72 | 97.01 | 4.04 | 0.0111 | 127 | 652.58 | 27.19 | 0.0745 | 182 | 4389.98 | 182.92 | 0.5011 |
| 17 | 14.42 | 0.6 | 0.0016 | 73 | 100.43 | 4.18 | 0.0115 | 128 | 675.59 | 28.15 | 0.0771 | 183 | 4544.8 | 189.37 | 0.5188 |
| 18 | 14.93 | 0.62 | 0.0017 | 74 | 103.97 | 4.33 | 0.0119 | 129 | 699.41 | 29.14 | 0.0798 | 184 | 4705.07 | 196.04 | 0.5371 |
| 19 | 15.45 | 0.64 | 0.0018 | 75 | 107.63 | 4.48 | 0.0123 | 130 | 724.08 | 30.17 | 0.0827 | 185 | 4870.99 | 202.96 | 0.556 |
| 20 | 16 | 0.67 | 0.0018 | 76 | 111.43 | 4.64 | 0.0127 | 131 | 749.61 | 31.23 | 0.0856 | 186 | 5042.77 | 210.12 | 0.5757 |
| 21 | 16.56 | 0.69 | 0.0019 | 77 | 115.36 | 4.81 | 0.0132 | 132 | 776.05 | 32.34 | 0.0886 | 187 | 5220.6 | 217.53 | 0.596 |
| 22 | 17.15 | 0.71 | 0.002 | 78 | 119.43 | 4.98 | 0.0136 | 133 | 803.41 | 33.48 | 0.0917 | 188 | 5404.7 | 225.2 | 0.617 |
| 23 | 17.75 | 0.74 | 0.002 | 79 | 123.64 | 5.15 | 0.0141 | 134 | 831.75 | 34.66 | 0.0949 | 189 | 5595.3 | 233.14 | 0.6387 |
| 24 | 18.38 | 0.77 | 0.0021 | 80 | 128 | 5.33 | 0.0146 | 135 | 861.08 | 35.88 | 0.0983 | 190 | 5792.62 | 241.36 | 0.6613 |
| 25 | 19.03 | 0.79 | 0.0022 | 81 | 132.51 | 5.52 | 0.0151 | 136 | 891.44 | 37.14 | 0.1018 | 191 | 5996.9 | 249.87 | 0.6846 |
| 26 | 19.7 | 0.82 | 0.0022 | 82 | 137.19 | 5.72 | 0.0157 | 137 | 922.88 | 38.45 | 0.1054 | 192 | 6208.38 | 258.68 | 0.7087 |
| 27 | 20.39 | 0.85 | 0.0023 | 83 | 142.02 | 5.92 | 0.0162 | 138 | 955.43 | 39.81 | 0.1091 | 193 | 6427.31 | 267.8 | 0.7337 |
| 28 | 21.11 | 0.88 | 0.0024 | 84 | 147.03 | 6.13 | 0.0168 | 139 | 989.12 | 41.21 | 0.1129 | 194 | 6653.97 | 277.25 | 0.7596 |
| 29 | 21.86 | 0.91 | 0.0025 | 85 | 152.22 | 6.34 | 0.0174 | 140 | 1024 | 42.67 | 0.1169 | 195 | 6888.62 | 287.03 | 0.7864 |
| 30 | 22.63 | 0.94 | 0.0026 | 86 | 157.59 | 6.57 | 0.018 | 141 | 1060.11 | 44.17 | 0.121 | 196 | 7131.55 | 297.15 | 0.8141 |
| 31 | 23.43 | 0.98 | 0.0027 | 87 | 163.14 | 6.8 | 0.0186 | 142 | 1097.5 | 45.73 | 0.1253 | 197 | 7383.04 | 307.63 | 0.8428 |
| 32 | 24.25 | 1.01 | 0.0028 | 88 | 168.9 | 7.04 | 0.0193 | 143 | 1136.2 | 47.34 | 0.1297 | 198 | 7643.41 | 318.48 | 0.8725 |
| 33 | 25.11 | 1.05 | 0.0029 | 89 | 174.85 | 7.29 | 0.02 | 144 | 1176.27 | 49.01 | 0.1343 | 199 | 7912.95 | 329.71 | 0.9033 |
| 34 | 25.99 | 1.08 | 0.003 | 90 | 181.02 | 7.54 | 0.0207 | 145 | 1217.75 | 50.74 | 0.139 | 200 | 8192 | 341.33 | 0.9352 |
| 35 | 26.91 | 1.12 | 0.0031 | 91 | 187.4 | 7.81 | 0.0214 | 146 | 1260.69 | 52.53 | 0.1439 | 201 | 8480.89 | 353.37 | 0.9681 |
| 36 | 27.86 | 1.16 | 0.0032 | 92 | 194.01 | 8.08 | 0.0221 | 147 | 1305.15 | 54.38 | 0.149 | 202 | 8779.97 | 365.83 | 1.0023 |
| 37 | 28.84 | 1.2 | 0.0033 | 93 | 200.85 | 8.37 | 0.0229 | 148 | 1351.18 | 56.3 | 0.1542 | 203 | 9089.59 | 378.73 | 1.0376 |
| 38 | 29.86 | 1.24 | 0.0034 | 94 | 207.94 | 8.66 | 0.0237 | 149 | 1398.83 | 58.28 | 0.1597 | 204 | 9410.14 | 392.09 | 1.0742 |
| 39 | 30.91 | 1.29 | 0.0035 | 95 | 215.27 | 8.97 | 0.0246 | 150 | 1448.15 | 60.34 | 0.1653 | 205 | 9741.98 | 405.92 | 1.1121 |
| 40 | 32 | 1.33 | 0.0037 | 96 | 222.86 | 9.29 | 0.0254 | 151 | 1499.22 | 62.47 | 0.1711 | 206 | 10085.54 | 420.23 | 1.1513 |
| 41 | 33.13 | 1.38 | 0.0038 | 97 | 230.72 | 9.61 | 0.0263 | 152 | 1552.09 | 64.67 | 0.1772 | 207 | 10441.2 | 435.05 | 1.1919 |
| 42 | 34.3 | 1.43 | 0.0039 | 98 | 238.86 | 9.95 | 0.0273 | 153 | 1606.83 | 66.95 | 0.1834 | 208 | 10809.41 | 450.39 | 1.234 |
| 43 | 35.51 | 1.48 | 0.0041 | 99 | 247.28 | 10.3 | 0.0282 | 154 | 1663.49 | 69.31 | 0.1899 | 209 | 11190.6 | 466.28 | 1.2775 |
| 44 | 36.76 | 1.53 | 0.0042 | 100 | 256 | 10.67 | 0.0292 | 155 | 1722.16 | 71.76 | 0.1966 | 210 | 11585.24 | 482.72 | 1.3225 |
| 45 | 38.05 | 1.59 | 0.0043 | 101 | 265.03 | 11.04 | 0.0303 | 156 | 1782.89 | 74.29 | 0.2035 | 211 | 11993.79 | 499.74 | 1.3692 |
| 46 | 39.4 | 1.64 | 0.0045 | 102 | 274.37 | 11.43 | 0.0313 | 157 | 1845.76 | 76.91 | 0.2107 | 212 | 12416.75 | 517.36 | 1.4174 |
| 47 | 40.79 | 1.7 | 0.0047 | 103 | 284.05 | 11.84 | 0.0324 | 158 | 1910.85 | 79.62 | 0.2181 | 213 | 12854.63 | 535.61 | 1.4674 |
| 48 | 42.22 | 1.76 | 0.0048 | 104 | 294.07 | 12.25 | 0.0336 | 159 | 1978.24 | 82.43 | 0.2258 | 214 | 13307.94 | 554.5 | 1.5192 |
| 49 | 43.71 | 1.82 | 0.005 | 105 | 304.44 | 12.68 | 0.0348 | 160 | 2048 | 85.33 | 0.2338 | 215 | 13777.25 | 574.05 | 1.5727 |
| 50 | 45.25 | 1.89 | 0.0052 | 106 | 315.17 | 13.13 | 0.036 | 161 | 2120.22 | 88.34 | 0.242 | 216 | 14263.1 | 594.3 | 1.6282 |
| 51 | 46.85 | 1.95 | 0.0053 | 107 | 326.29 | 13.6 | 0.0372 | 162 | 2194.99 | 91.46 | 0.2506 | 217 | 14766.09 | 615.25 | 1.6856 |
| 52 | 48.5 | 2.02 | 0.0055 | 108 | 337.79 | 14.07 | 0.0386 | 163 | 2272.4 | 94.68 | 0.2594 | 218 | 15286.81 | 636.95 | 1.7451 |
| 53 | 50.21 | 2.09 | 0.0057 | 109 | 349.71 | 14.57 | 0.0399 | 164 | 2352.53 | 98.02 | 0.2686 | 219 | 15825.9 | 659.41 | 1.8066 |
| 54 | 51.98 | 2.17 | 0.0059 | 110 | 362.04 | 15.08 | 0.0413 | 165 | 2435.5 | 101.48 | 0.278 | 220 | 16384 | 682.67 | 1.8703 |
| 55 | 53.82 | 2.24 | 0.0061 | 111 | 374.81 | 15.62 | 0.0428 | 166 | 2521.38 | 105.06 | 0.2878 | 221 | 16961.78 | 706.74 | 1.9363 |
| 56 | 55.72 | 2.32 | 0.0064 |  |  |  |  |  |  |  |  |  |  |  |  |

**Table S3**: List of wavelets with their frequencies used in the wavelet power transformation

**Table S4**: Continuous periods of meteorological series in 1-hour resolution without missing data used as an input into the wavelet power transformation

| **Site** | **Species** | **Continuous period** | | **Missing 1-hour timesteps replaced by the long-term median** | |
| --- | --- | --- | --- | --- | --- |
|  |  | **Temperature** | **VPD** | **Temperature** | **VPD** |
| DAV  SCH | *Picea abies* | 01/2015 – 05/2019 | 01/2015 – 05/2019 | 1 | 5 |
| SUR  SCU | *Pinus sylvestris* | 01/2015 – 10/2018 | 01/2015 – 10/2018 | 0 | 0 |
| VIL  EL* | *Acer pseudoplatanus* | 01/2015 – 12/2020 | 09/2016 – 12/2020 | 0 | 2 |
|  | *Fagus sylvatica* |  |  |  |  |
|  | *Quercus robur* |  |  |  |  |
| EL* | *Carpinus betulus* | 01/2015 – 02/2020 | Substantiated from *Acer, Fagus, Quercus* | 0 | 2 |

* Since sites ELM and ELU are located close to each other, there is only one installation of meteorological sensors relevant for both sites (EL).

*Site codes: ELM = Eldena managed; ELU = Eldena unmanaged; VIL = Vilm; DAV = Davos; SCH = Schmitten; SCU = Scuol; SUR = Surava*


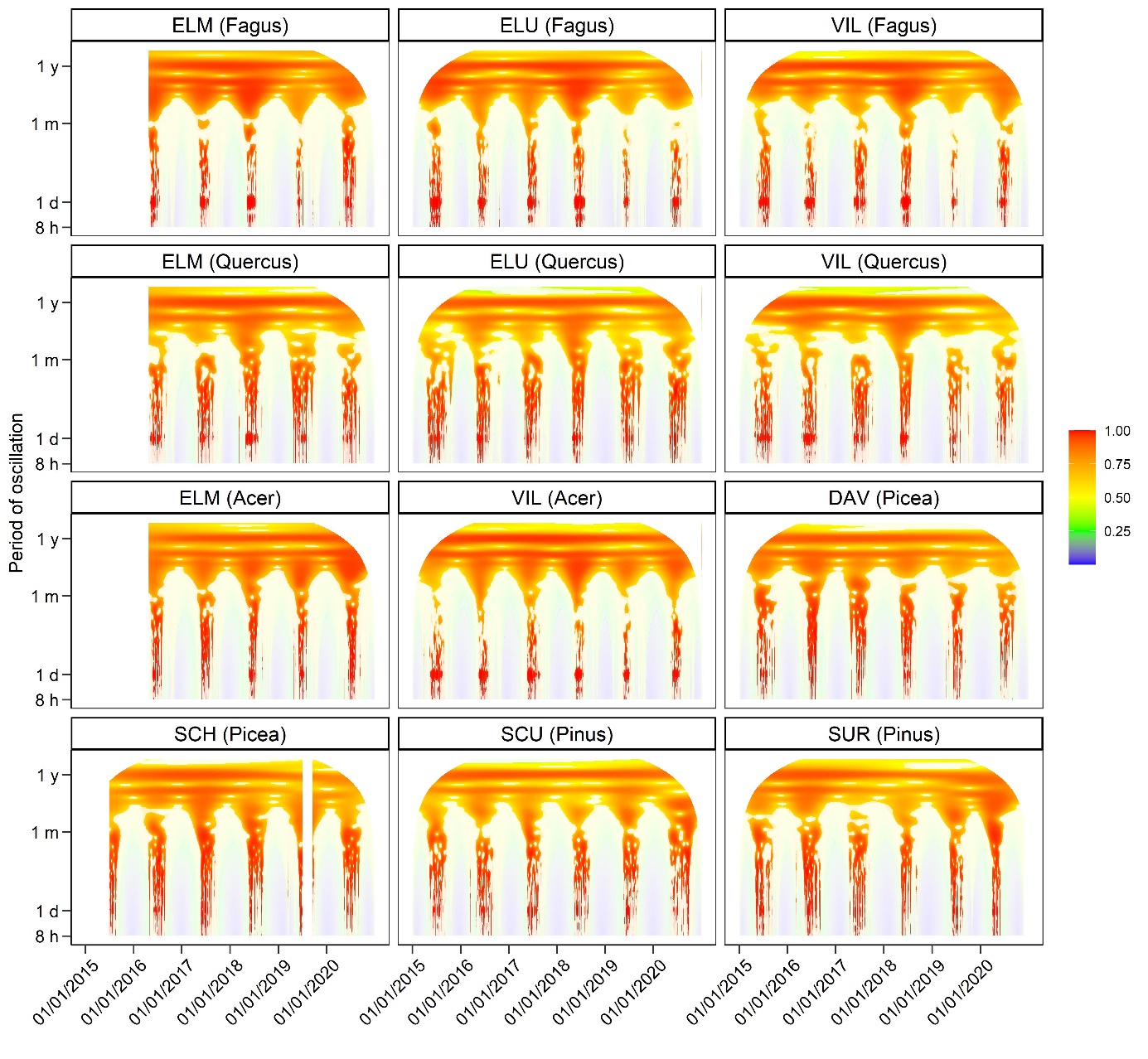


**Figure S1:** The wavelet power spectrum of periodic oscillations in radial growth rate calculated for individual sites and sufficiently-long continuous segments without missing data. X-axes of the matrix indicate the 1-hour timestep and Y-axes of the matrix indicate the period of wavelet oscillation (h = hour, d = day, m = synodic month ≈ 29.53 d, y = year). The colour gradient represents the standardised (0-1) power of the wavelet with red and blue colours indicating oscillations with the highest and lowest power for the given species and site, respectively. The colour of pixels with an estimated p-value > 0.05, i.e., non-significant wavelet power, is semitransparent.

*Site codes: ELM = Eldena managed; ELU = Eldena unmanaged; VIL = Vilm; DAV = Davos; SCH = Schmitten; SCU = Scuol; SUR = Surava*


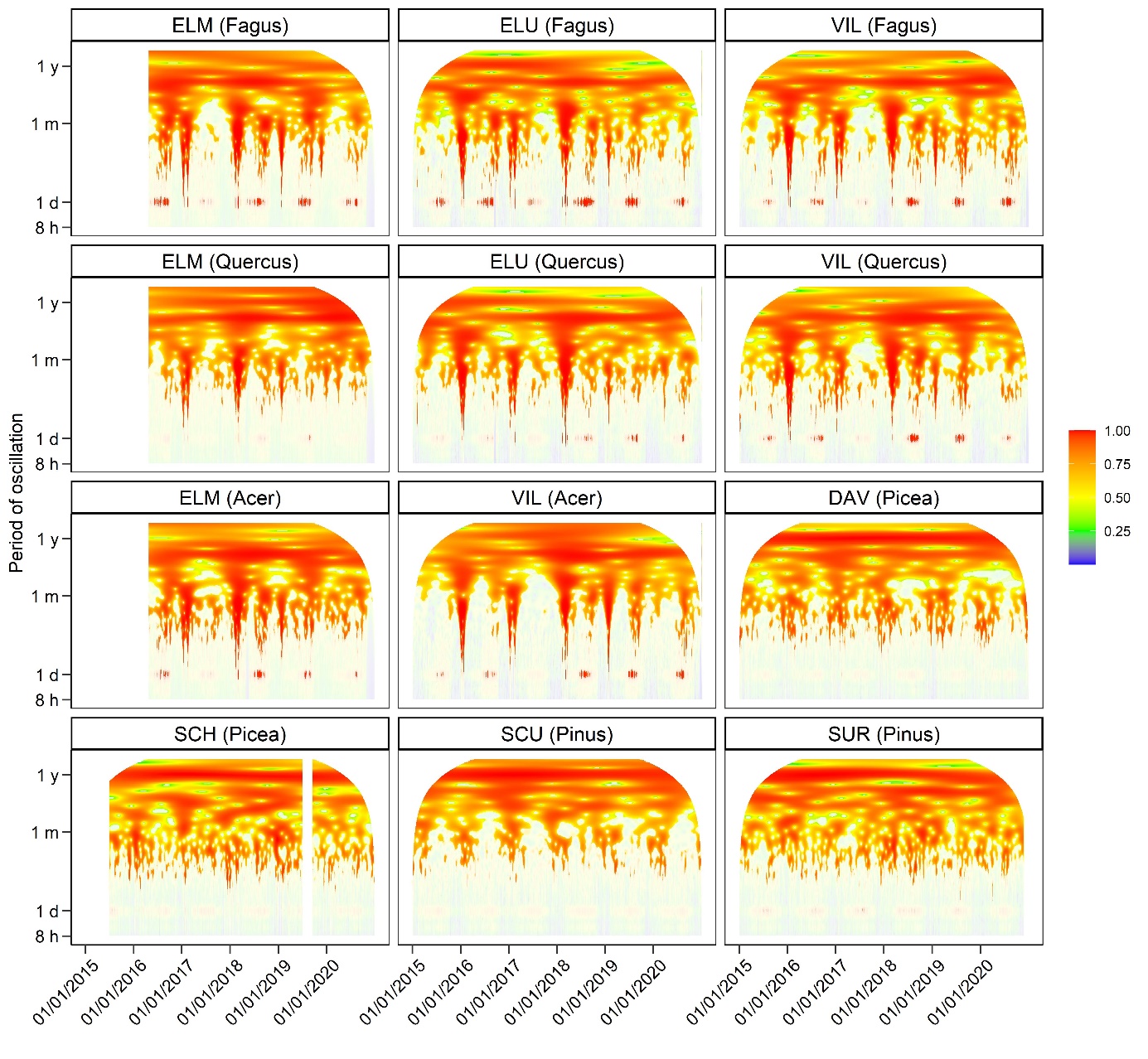


**Figure S2:** The wavelet power spectrum of periodic oscillations in tree water deficit calculated for individual sites and sufficiently-long continuous segments without missing data. X-axes of the matrix indicate the 1-hour timestep and Y-axes of the matrix indicate the period of wavelet oscillation (h = hour, d = day, m = synodic month ≈ 29.53 d, y = year). The colour gradient represents the standardised power of the wavelet with red and blue colours indicating oscillations with the highest and lowest power for given species and site, respectively. The colour of pixels with an estimated p-value > 0.05, i.e., non-significant wavelet power, is semitransparent.

*Site codes: ELM = Eldena managed; ELU = Eldena unmanaged; VIL = Vilm; DAV = Davos; SCH = Schmitten; SCU = Scuol; SUR = Surava*


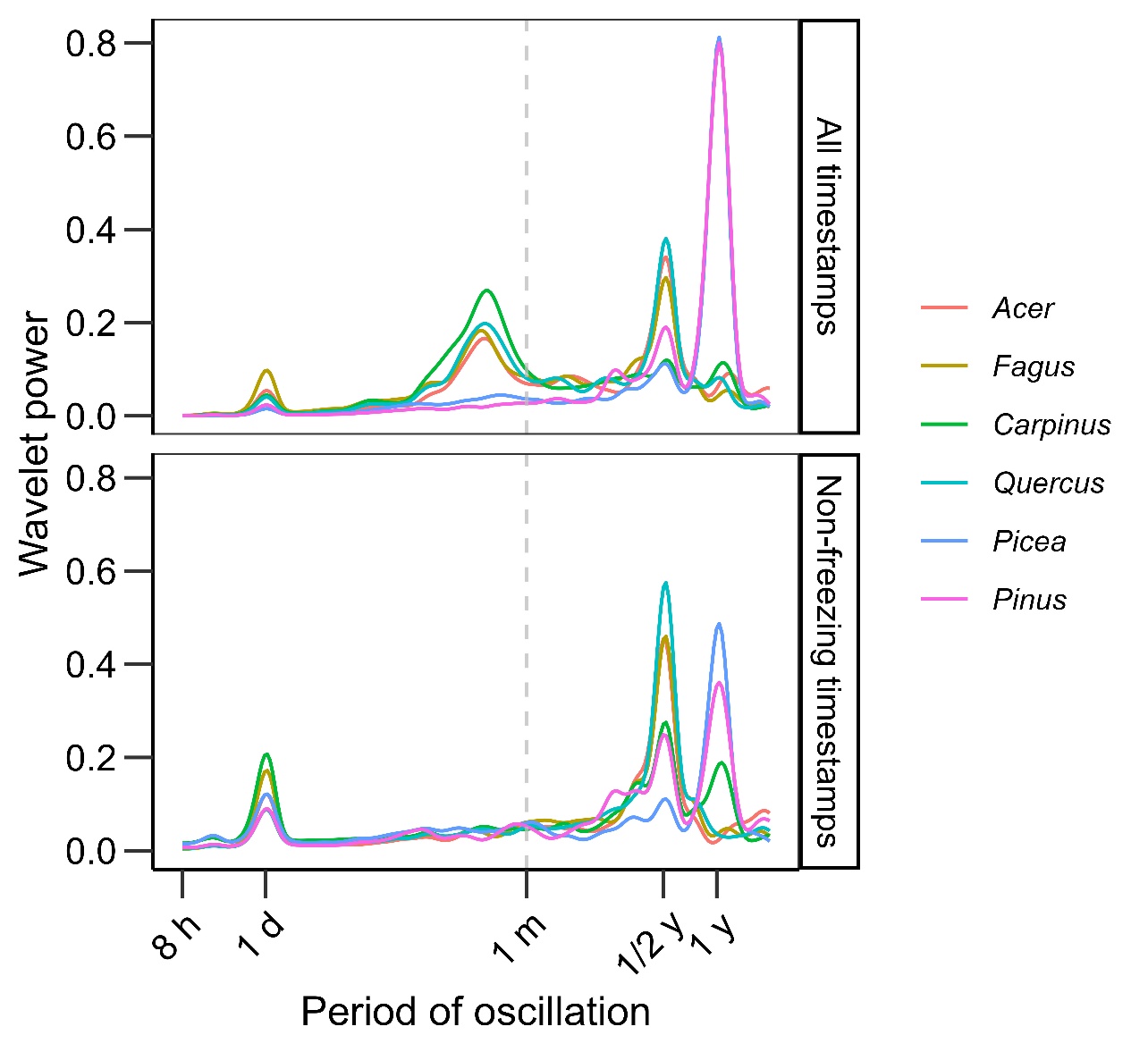


**Figure S3:** Mean power of wavelets in tree water deficit for original series (top row) and series with equalised tree water deficit during timestamps with air temperature below 0 °C to exclude irregular stem-freezing events (bottom row). h = hour, d = day, m = synodic month ≈ 29.53 d, y = year


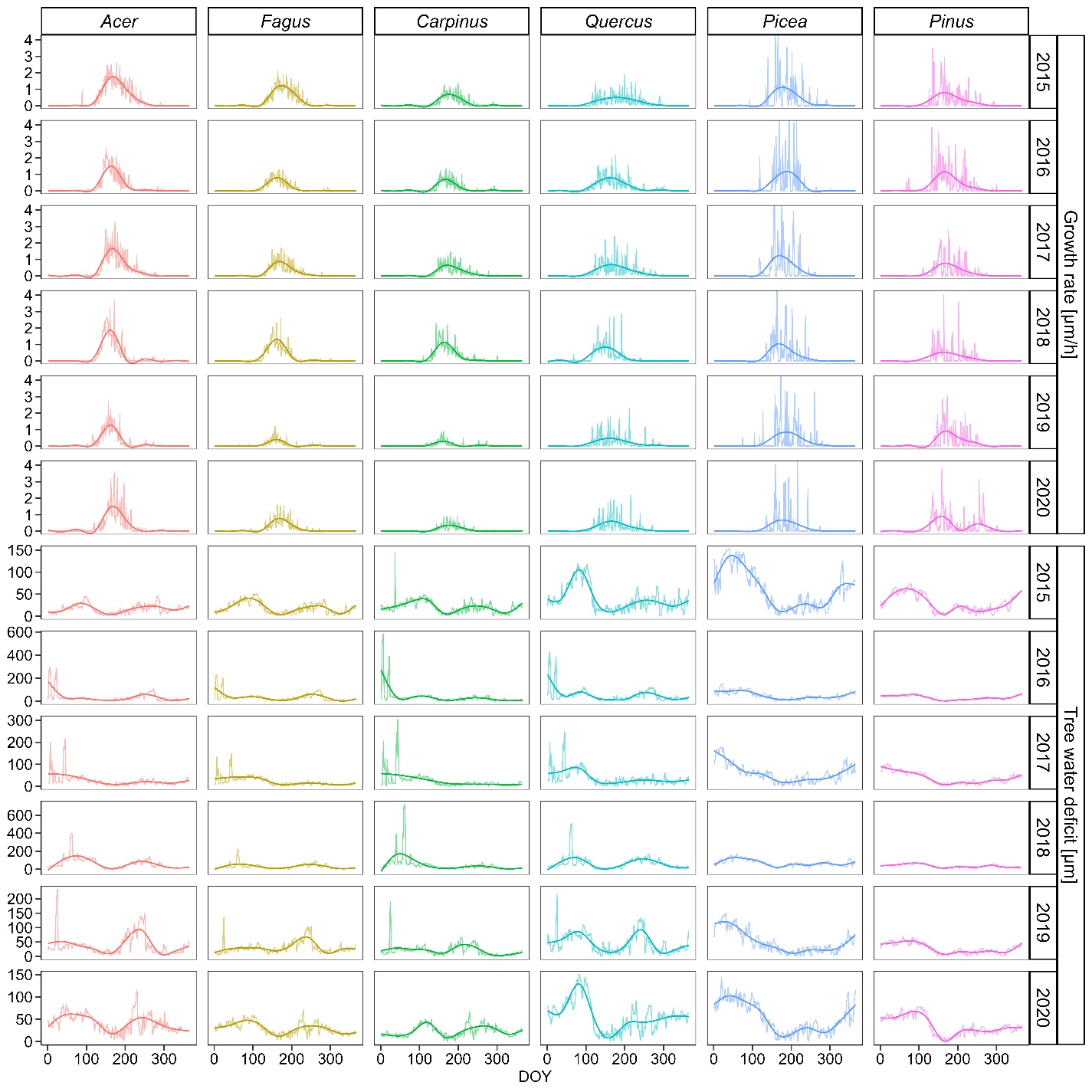


**Figure S4:** Mean annual course of radial growth rate and tree water deficit of individual trees averaged at the species level. Thin and thick lines indicate raw data and their annual trend approximated by a generalised additive model, respectively. The data were aggregated from 1-hour series to daily resolution (x-axes) to improve visual clarity of charts. To improve the comparison between species, values of tree water deficit of conifers were multiplied by 0.2. Note different scales of y-axes for tree water deficit due to significant variability in mean tree water deficit between years. DOY = day of the year.


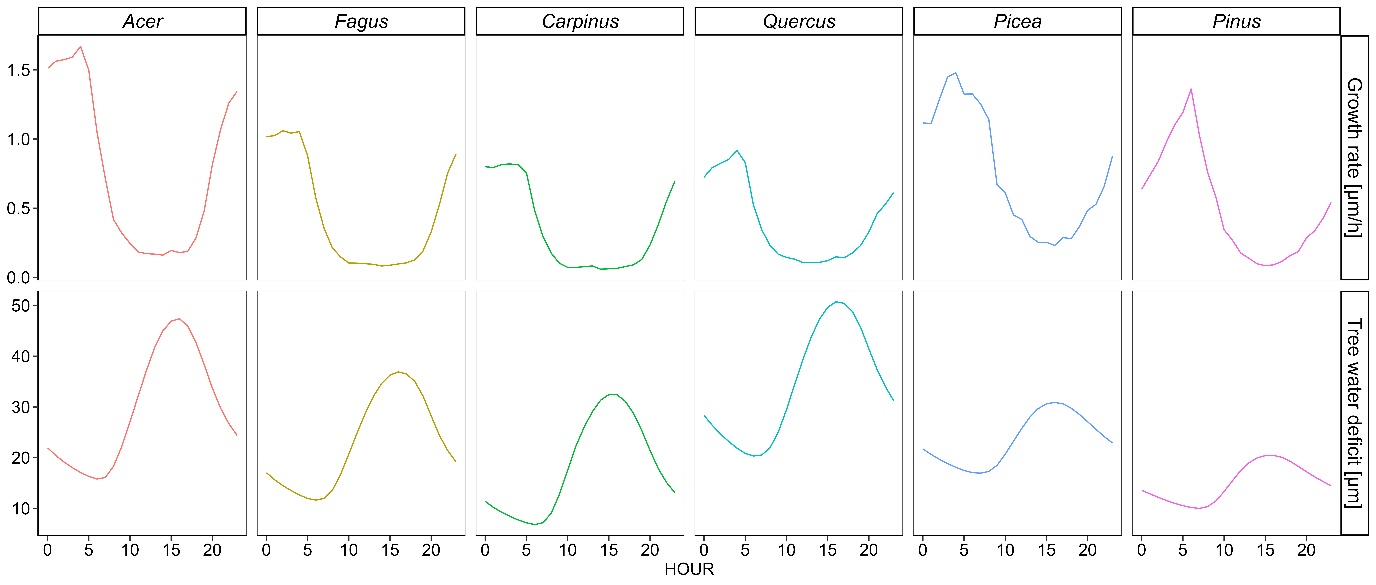


**Figure S5**: Mean values of growth rate and tree water deficit of individual species averaged for hours of the day during summer seasons (June-August) between January 2015 and December 2020. Values of tree water deficit of conifers were multiplied by 0.2 to facilitate comparison between species.


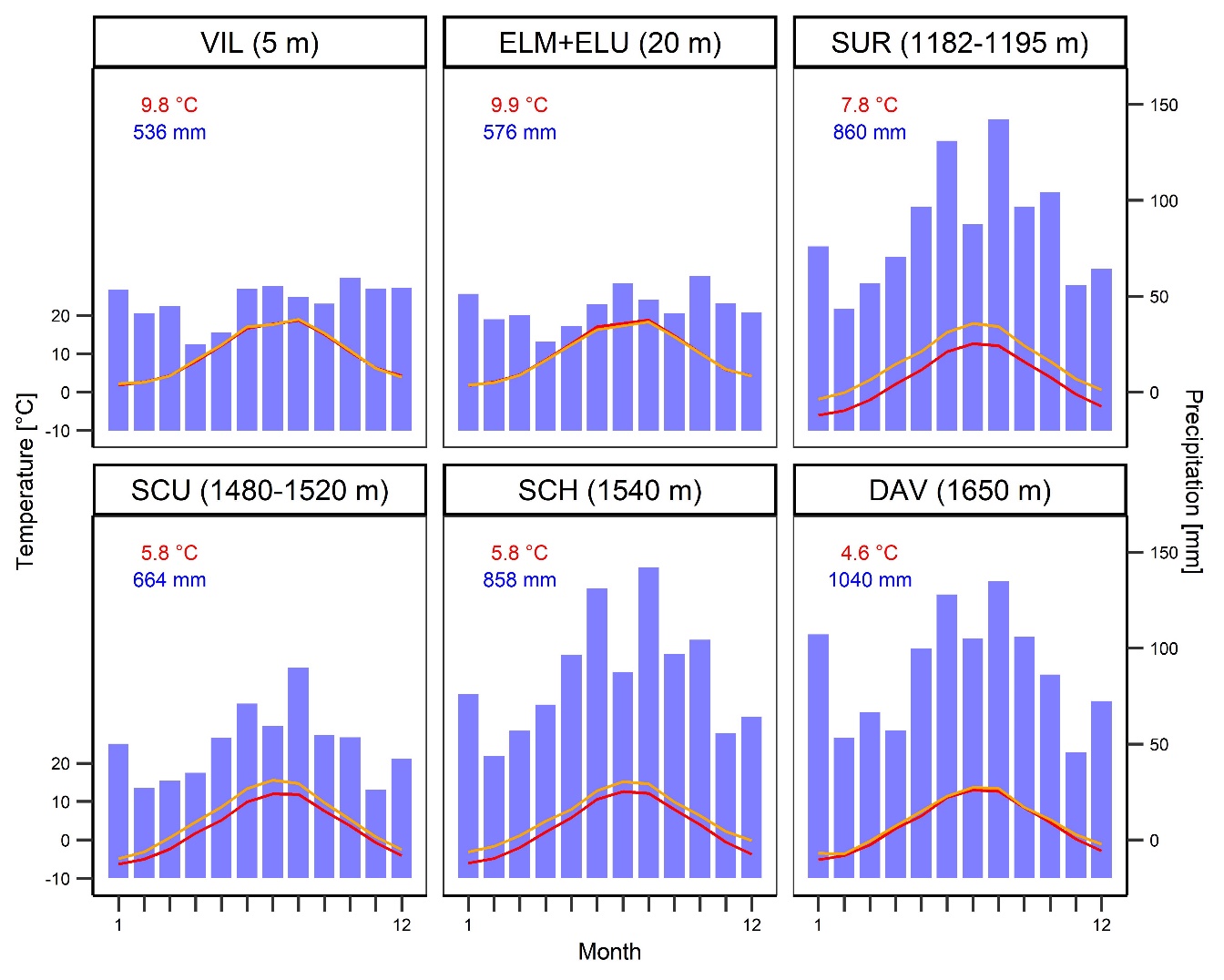


**Figure S6**: Climatic diagrams for dendrometer monitoring sites based on the period between 1^st^ January 2015 and 31^st^ December 2020. Lines and blue bars show the mean monthly temperature and total monthly precipitation, respectively. The red lines show the mean temperature according to E-OBS 25.0. gridded climatic surface with a resolution of 0.1x0.1° interpolated from a network of meteorological stations, orange lines are based on our *in-situ* meteorological measurements in the forest at each plot. Precipitation data were taken from E-OBS 25.0. database. Numbers in the top-left corner indicate the mean annual temperature and total annual precipitation.

*Site codes: ELM = Eldena managed; ELU = Eldena unmanaged; VIL = Vilm; DAV = Davos; SCH = Schmitten; SCU = Scuol; SUR = Surava*
